# Co-Occurrence of Two Allelic Variants of CYP51 in *Erysiphe necator* and Their Correlation with Over-Expression for DMI Resistance

**DOI:** 10.1371/journal.pone.0148025

**Published:** 2016-02-03

**Authors:** Lynn Esther E. Rallos, Anton B. Baudoin

**Affiliations:** Department of Plant Pathology, Physiology and Weed Science, College of Agriculture and Life Sciences, Virginia Tech, Blacksburg, Virginia, United States of America; University of California Davis, UNITED STATES

## Abstract

Demethylation inhibitors (DMIs) have been an important tool in the management of grapevine powdery mildew caused by *Erysiphe necator*. Long-term, intensive use of DMIs has resulted in reduced sensitivity in field populations. To further characterize DMI resistance and understand resistance mechanisms in this pathogen, we investigated the *cyp51* sequence of 24 single-spored isolates from Virginia and surrounding states and analyzed gene expression in isolates representing a wide range of sensitivity. Two *cyp51* alleles were found with respect to the 136^th^ codon of the predicted *En*CYP51 sequence: the wild-type (TAT) and the mutant (TTT), which results in the known Y136F amino acid change. Some isolates possessed both alleles, demonstrating gene duplication or increased gene copy number and possibly a requirement for at least one mutant copy of CYP51 for resistance. *Cyp51* was over-expressed 1.4- to 19-fold in Y136F-mutant isolates. However, the Y136F mutation was absent in one isolate with moderate to high resistance factor. Two additional synonymous mutations were detected as well, one of which, A1119C was present only in isolates with high *cyp51* expression. Overall, our results indicate that at least two mechanisms, *cyp51* over-expression and the known target-site mutation in CYP51, contribute to resistance in *E*. *necator*, and may be working in conjunction with each other.

## Introduction

Commercial varieties of the European grape species *Vitis vinifera* are susceptible to powdery mildew caused by *Erysiphe necator* [1–3]. Disease control relies heavily on the use of protective and systemic fungicides in conjunction with aggressive cultural practices. Among the widely applied fungicides against *E*. *necator* are demethylation inhibitors (DMIs). DMIs inhibit the cytochrome P450 eburicol 14α-demethylase (P450_14DM_) of *E*.*necator*, encoded by the *cyp51* gene [4, 5]. This demethylase acts as a key enzyme for fungal sterol biosynthesis [5, 6].

The first DMI labeled for use in grapes in the Unites States was triadimefon, first introduced in 1982 [7]. Other DMIs such as fenarimol, myclobutanil, triflumizole and tebuconazole followed suit. In 2009 and 2010, tetraconazole and difenoconazole were registered, respectively. Most of these belong to the triazole chemical family but fenarimol is a pyrimidine. The DMIs now constitute the largest group of fungicides for grapevine powdery mildew control in the United States. Additional DMIs have been registered in other areas of the world [5]. To alleviate the selection pressure towards fungicide resistance and to improve disease control, DMIs are often used as rotational or mixture partners with non-cross-resistant chemistries [8]. However, the intensive use of DMIs has led to outbreaks of pathogen resistance. Early US reports were made for *E*. *necator* populations in California [9–11], New York [12, 13], and Ontario [14]. Reduced sensitivity to DMIs has been reported for Virginia and neighboring states [15]. DMI resistance and sensitivity shifts have also been observed in *E*. *necator* populations in Europe and Australia [16–19]. However, complete loss of efficacy has not yet been reported, possibly due to the association of a fitness cost as reported for some fungi [20] and/or the quantitative nature of resistance [21].

Resistance to DMIs has been attributed to several mechanisms [22]. Target site mutations that generate amino acid changes in the demethylase enzyme have been identified in a number of fungi [23]. The *Encyp51* protein inferred to be 524 amino acids in length is encoded by the ~1.6 kb-coding region of the ~1.7 kb gene with six highly conserved amino acid domains (CR1 to 6) that are highly typical of cytochrome P450 and CYP51s [24]. An amino acid change from tyrosine to phenylalanine at position 136 (Y136F) was found in triadimenol-resistant isolates that were moderately to highly resistant [25], and in isolates resistant to various DMIs [15, 26]. DMI resistance in other organisms has also been attributed to (i) other CYP51 mutations in combination or not with Y136F [27–35]; (ii) increased *cyp51* expression [36–40]; and (iii) increased efflux pump activity or over-expression of ABC transporters that can confer multi-drug resistance [22, 41–44]. A combination of mechanisms, target-site mutation and CYP51 over-expression, has been documented for *E*. *necator* [45].

Cross-resistance to fungicides with the same mode of action can occur. Different mechanisms may allow for differential selection of resistant strains. For example, the I381V mutation in CYP51 of *M*. *graminicola* was selected by tebuconazole and difenoconazole, but less aggressively or not at all by other triazoles [35]. Interestingly, a group of isolates in our collection having high resistance to tebuconazole and myclobutanil have variable sensitivity to the other DMIs such as triadimefon and fenarimol [15]. This may be due to the inherent activity of the fungicides but may also be due to molecule-specific mechanisms [22]. To determine if DMI-specific mechanisms exist for *E*. *necator*, we further investigated the *cyp51* gene by sequence analysis of 24 isolates with various degrees of sensitivity to tebuconazole, fenarimol and myclobutanil and determined *cyp51* over-expression in representative isolates. We particularly wanted to find out whether or not the variation in DMI sensitivity may be largely controlled by the single point mutation (Y136F) as shown previously in European strains [25] and in some of our resistant isolates [15]. Our findings revealed that CYP51 over-expression is closely associated with Y136F. We further discovered the co-occurrence of non-mutated and mutated CYP51 sequences in highly resistant isolates and its association with high DMI resistance factors. This finding supports a correlation of *En*CYP51 copy number variation and target-site mutation [45]. This research was part of a doctoral dissertation by the senior author [46]. After our experiments were finished, papers by Jones et al. [45] and Frenkel et al. [26] confirmed copy number variation of *EnCYP51* and association of Y136F and *cyp51* over-expression to DMI resistance. Neither of the other two studies reported our finding of the co-occurrence of *cyp51* wild-type and mutant alleles.

## Materials and Methods

### Culture preparation and DNA extraction

Twenty-four laboratory-maintained isolates for *cyp51* sequencing, 3 fresh isolates from the field for verifying wild-type *cyp51* at codon 136 (Y136), and another 24 fresh isolates for checking the SNP genotyping method were used in this study. The locations and date of collection for these isolates are summarized in Table 1. Fungicide-free and powdery mildew-free grape leaves used for culture transfers and maintenance were taken from plants (*Vitis vinifera* cv. Chardonnay) maintained in the greenhouse. After harvest, leaves were immediately surface-disinfested with 10% commercial bleach for 1 minute, rinsed three times with sterilized distilled water and plated onto 1.5% water agar, one leaf per plate [15].

**Table 1:** Sources of *Erysiphe necator* isolates used in this study and their DMI sensitivities expressed as EC_50_ to three demethylation inhibitors.

Isolate	Location, State	Coordinates	Collection year and month	EC_50_		
				Tebuconazole	Myclobutanil	Fenarimol
BLP1	Blacksburg, VA	37.242, -80.404	2005 Sep	0.03	0.06	0.005
BLP4	Blacksburg, VA	37.242, -80.404	2006 Jul	0.03	0.34	0.01
MVP9	Nathalie, VA	36.908, -79.109	2007 Sep	0.02	0.13	0.03
PBP1	New Kent, VA	37.529, -77.075	2006 Oct	0.03	0.07	0.02
SCCP4	Dobson, NC	36.382, -80.720	2006 Oct	0.05	0.008	0.03
SNP1	White Hall, VA	38.10, -78.78	2010 Jul	0.03	nt	nt
SNP3	White Hall, VA	38.10, -78.78	2010 Jul	0.02	nt	nt
FH9-1	Glasgow, VA	37.696, -79.487	2009 Oct	0.01	nt	nt
VAHP6	Smith River, VA	36.734, -80.194	2006 Oct	0.60	4.40	0.34
BXP1A	Bloxom, VA	37.820, -75.623	2006 Jun	0.17	0.85	0.11
GRP15	Shiloh, VA	38.198, -77.069	2007 Aug	0.46	0.003	0.29
GRP18	Shiloh, VA	38.198, -77.069	2007 Aug	0.45	7.87	0.18
IVP3	Washington, VA	38.150, -77.001	2007 Aug	0.04	0.03	0.03
IVP11	Washington, VA	38.150, -77.001	2007 Aug	1.68	nt	0.26
MDMRP5	Upper Marlboro, MD	38.860, -76.780	2006 Sep	0.56	4.60	0.11
MDMRP7	Upper Marlboro, MD	38.860, -76.780	2006 Sep	0.65	9.07	0.40
PRP7	Leon, VA	38.436, -78.154	2007 Aug	1.18	13.83	0.25
ROP14	Raphine, VA	37.943, -79.242	2007 Aug	0.70	7.00	0.19
SUP13-2	Stanardsville, VA	38.326, -78.453	2005 Aug	0.28	4.57	0.08
VAHP1	Smith River, VA	36.734, -80.194	2006 Oct	0.25	0.73	0.08
AMP1	Afton, VA	38.008, -78.852	2007 Jul	1.66	3.02	0.98
IVP4	Washington, VA	38.150, -77.001	2007 Aug	0.58	7.08	0.48
JRP3	Montpelier, VA	37.836, -77.703	2006 Oct	0.75	8.98	0.74
VAHP4	Smith River, VA	36.734, -80.194	2006 Oct	0.64	3.57	0.54
JRP1	Montpelier, VA	37.836, -77.703	2006 Oct	0.83	11.50	0.14
JRP4	Montpelier, VA	37.836, -77.703	2006 Oct	0.97	8.52	0.45
JWP1	Manheim, PA	40.172, -76.438	2006 Oct	3.50	6.00	1.33
AF isolates	Boones Mill, VA	37.101, -80.016	2010 Jun-Sep	nt	nt	nt
RO isolates	Raphine, VA	37.943, -79.242	2010 Jul-Aug	nt	nt	nt
SC isolates	Dobson, NC	36.382, -80.720	2010 Aug-Sep	nt	nt	nt

nt = not tested

To ensure purity of laboratory isolates, a single spore chain was carefully picked from a growing culture and transferred to the center of a grape leaf in a water agar plate. This was done using a sterile acupuncture needle while viewing the transfer process under a stereomicroscope placed in a laminar-flow hood. The single chain-inoculum was allowed to grow until single spore chains could be picked easily (about 8–12 days). The single-chain method was repeated for each isolate. On the third transfer, multiple chains of conidia for each isolate were deposited on a leaf in a water agar plate and incubated in a clear plastic box under a 12 hr-light regime. At this point, the cultures were considered pure.

For new field (fresh) isolates, infected leaf samples were first gently rubbed onto clean grape leaves. Two leaves were used for each leaf sample to increase our chance of getting powdery mildew growth. Once powdery mildew growth was seen, a single chain of spores was picked from one of the two leaves, and this process was repeated. On the third transfer, pure cultures were generated as described earlier for laboratory samples. Multiple culture plates were prepared per isolate for DNA extraction and fungicide sensitivity assay.

For DNA extraction, fungal material (primarily spores and some conidiophore and hyphal fragments) from pure cultures was scraped into a 2-ml conical tube containing sterile 0.005% Tween 20 water (STW). At least three sporulating leaves were used for DNA extraction. To extract DNA, the Biosprint 15 DNA Plant Kit (Qiagen) was used following the protocol in Colcol et al. [15], performed with the Biosprint 15 workstation (Qiagen).

### Fungicide sensitivity assay

Isolates for *cyp51* sequencing came from cultures that had been maintained in the laboratory on fungicide-free grape leaves for over two years by sequential transfer to new leaves every two to three weeks. Pure cultures for each isolate were prepared as described in the previous section. Fungicide sensitivity assays were conducted as close as possible to the time of DNA extraction for *cyp51* sequencing. Sensitivities to myclobutanil, tebuconazole and fenarimol were determined by detached-leaf bioassays. Stock solutions of tebuconazole (Bayer Crop Science LP, Research Triangle Park, NC), myclobutanil (Dow Agrosciences LLC, Indianapolis, IN) and fenarimol (Gowan Company, Yuma, AZ) were prepared in acetone from technical grade fungicides and stored at -10 to -12°C. A 3x-serial dilution scheme was used as follows depending on the isolate and DMI fungicide: 0.001–0.1, 0.003–0.3, 0.01–1, 0.03–0.3, 0.1–1, 0.3–3, 1–10, or 3–30 mg liter^-1^. Bioassays were done by a point-inoculation method where six spore chains from each culture were deposited at equal distance from each other on the adaxial side of a leaf on 0.7% water agar (two leaves per fungicide treatment). Leaves were shaken in fungicide solution as described in Colcol et al. [15], then incubated at 25°C under a 12-hr light regime for 6 to 8 days. Relative growth was calculated from the mean colony diameters as a percentage of the mean diameter of the control (no fungicide treatment). The relative diameter at different treatments was regressed on natural log-transformed fungicide concentration. From the linear section of the regression, the effective concentration to inhibit growth by 50% (EC_50_) was calculated in Microsoft Excel as described in Colcol et al. [15]. The resistance factor (RF) was calculated for each isolate as EC_50_ for the fungicide divided by the corresponding mean EC_50_ of the sensitive sub-group consisting of three isolates (BLP1, BLP4, MVP9) taken from unexposed populations (locations with no known fungicide application). These isolates were part of the larger sensitive sub-group reported by Colcol et al. [15]. Mean EC_50_ for our sensitive sub-group was calculated as 0.03 mg liter^-1^ for tebuconazole, 0.18 mg liter^-1^ for myclobutanil and 0.02 mg liter^-1^ for fenarimol. Isolates with RF<3 were regarded as sensitive; 3<RF<10 as weakly resistant; and RF ≥10 as resistant.

### *Cyp51* sequence analysis

Three primer sets were designed using Primer 3 [47] based on the *Encyp51* sequence (GenBank accession no. U72657) [24] to generate three overlapping sequences from the 24 laboratory-maintained isolates with different DMI resistance profiles. Primer sequences are summarized in Table 2 while *cyp51*-genotype and resistance phenotype of isolates are shown in Table 3. The 20 μl-PCR reaction consisted of 0.2 μM of each primer, 1X iProof HF Master Mix (Biorad, Hercules, CA, USA) and 10 to 50 ng of template DNA. The PCR reactions were performed in the Eppendorf Mastercycler (Eppendorf, Hamburg, Germany) with the following cycling conditions: initial denaturation at 98°C for 30 s, then 35 cycles of denaturation at 98°C for 10 s, annealing at 59°C (primers F1/R900), 52°C (primers F502/R1288) or 55°C (primers F1300/R1300) for 30 s, and extension at 72°C for 1 min. The final extension was set at 72°C for 5 min. The fourth primer pair, L/R419 (Table 2), was developed to amplify an even shorter region (~0.58kb) encompassing the 136^th^ position (nt 419–1000). This set was primarily intended to double-check the 136^th^ codon of four resistant isolates (AMP1, JRP3, IVP4, VAHP6; Table 3). Three additional isolates considered to be wild-type (SNP1, SNP3, FH9-1; Table 3) were included in this repeat sequencing. These isolates were collected from wild grapes. The PCR conditions for L/R419 were the same as the first sequencing protocol except for the annealing temperature which was modified to 53°C. Five microliters of each PCR product were cleaned enzymatically with shrimp alkaline phosphatase (USB, Affymetrix, Inc., Cleveland, OH, USA) and Exonuclease I (USB, Affymetrix, Inc., Cleveland, OH, USA) according to manufacturer’s protocol. Cleaned products were submitted for direct sequencing to the University of Chicago Sequencing Facility. Assembly of forward and reverse sequences to generate individual and consensus sequences, and amino acid translation were done using Lasergene 10 Core Suite (DNASTAR, Inc.). Amino acid sequences were compared to the predicted sequence for CYP51 (UniProtKB/Swiss-Prot Accession no. O14442) [24]. The consensus sequence harboring Y136F was designated mutant, while the sequence lacking the mutation was designated wild-type.

**Table 2:** Primer information for various PCR amplifications in the study of DMI resistance mechanisms in *Erysiphe necator*.

Primer	Sequence (5’→3’)	Application
L1	TTGTCGACCCCCAAGACTAC	*cyp51* sequencing, amplifies section 37–1700, ~1.3 kb amplicon
R900	GACTTGACGCTCCTGTGCTA	
L502	CGCCGAAGAGATTTACACTA	*cyp51* sequencing, amplifies section 405–1238, ~0.97 kb amplicon
R1000	GATCCCATTTGAGAGGGTCT	
L1300	CATGGAAGAGTTGTATGAGGAACA	*cyp51* sequencing, amplifies section 1097–1800, ~0.76 kb amplicon
R1300	CAATTCTTCTAACCCTAACACCTG	
L419	CAGTCTATCTGGGACTTCAAGG	*cyp51* sequencing/verifying codon 136, amplifies section 419–1000, ~0.58 amplicon
R419	AACAGTTCTTTGGGCATGAT	
FS*cyp51* RS*cyp51*	ACTAATTTAACAACTCCGGTCTTTGGA ACTCGACCATTTACGGACCTTTTT	*cyp51*-specific forward and reverse primers for SNP genotyping
Probe 1	VIC-TTGGACAATCAAATACAAC	mutant allele-specific and wild-type allele-specific probes, SNP genotyping
Probe 2	FAM-TTTGGACAATCATATACAAC	
FG*cyp51*	CATGCGCGAGATCGTTCAC	*cyp51*-forward and *cyp51*-reverse primers for gene expression analysis
RG*cyp51*	CAGAAATGGTTTGCCGAAAGCA	
FG*tub*	TGATTGTCCAAATTCCAAACTCATGGA	*tub*-forward and *tub*-reverse primers for gene expression analysis
RG*tub*	AGGAATGGAACGCTTCAATGGT	
P*cyp51*	FAM-AAGAGCCGTTTTCATAAACTTT	*Encyp51* probe, for gene expression analysis
P*tub*	FAM-CCAATGCGGAAATCAA	*Entub* probe, for gene expression analysis

**Table 3:** DMI resistance phenotype and *cyp51* sequence information for *Erysiphe necator* isolates with various DMI sensitivities.

Isolate	RF ^a^			136^th^ Codon in *Cyp51*			Other SNPs		Mean RQ^g^
	Teb	Myc	Fen	Sequencing^b^	Repeat sequencing^c^	Genotyping^d^	nt 1119^e^	nt 1170^f^	
BLP1	1	0.4	0.3	TAT	nt	wild-type	A	G	1.00e
BLP4	1	3	0.7	TAT	TAT	wild-type	A	G	nt
MVP9	1	1	2	TAT	nt	wild-type	A	A	1.10e
PBP1	1	0.6	1	TAT	nt	wild-type	A	A	1.34e
SCCP4	2	0.05	2	TAT	TAT	wild-type	A	A	nt
SNP1	1	nt	nt	TAT	TAT	wild-type	nt	nt	nt
SNP3	0.6	nt	nt	TAT	TAT	wild-type	nt	nt	nt
FH9-1	0.4	nt	nt	TAT	TAT	nt	nt	nt	nt
VAHP6	24	33	20	TAT	TAT	mix/wild-type	C	G	nt
BXP1A	7	6	7	TTT	TTT	pure mutant	A	A	6.03d
GRP15	12	0.02	17	TTT	nt	pure mutant	A	A	nt
GRP18	18	59	11	TTT	TTT	pure mutant	A	A	nt
IVP3	2	0.2	2	TTT	nt	pure mutant	A	A	1.40e
IVP11	66	nt	15	TTT	nt	pure mutant	A	A	nt
MDMRP5	22	35	7	TTT	nt	pure mutant	A	A	10.98c
MDMRP7	25	68	24	TTT	TTT	pure mutant	A	A	12.92b
PRP7	46	104	15	TTT	nt	pure mutant	A	A	nt
ROP14	27	53	11	TTT	TTT	pure mutant	A	A	nt
SUP13-2	11	34	5	TTT	nt	pure mutant	A	A	nt
VAHP1	10	6	5	TTT	nt	pure mutant	A	A	1.85e
AMP1	65	23	59	TAT	TWT	mix	C	R	12.06c
IVP4	23	53	29	TAT	TWT	mix	C	R	10.73bc
JRP3	29	68	44	TAT	TWT	mix	C	R	nt
VAHP4	25	27	32	TWT	TWT	mix	C	R	11.58bc
JRP1	32	87	8	TWT	nt	mix	C	R	nt
JRP4	38	64	27	TWT	TWT	mix	C	R	18.85a
JWP1	137	45	79	TWT	nt	mix	C	R	nt

nt–not tested; cultures were either lost or isolates not selected for assays

^a^ Resistance factor from EC_50_ mean of three assays per culture; Teb = tebuconazole; Myc = myclobutanil; Fen = fenarimol

^b^ Corresponding to codon 136; TAT = tyrosine (Y), TTT = phenylalanine (F), TWT = double peak for middle nucleotide in sequence chromatograph

^c^ Repeat sequencing with primer pair L419/R419

^d^ Genotyping at the 136^th^ codon: wild-type = TAT; pure mutant = TTT; mix = TTT/TAT or TWT

^e^ Nucleotide 1119 displaying synonymous mutation at codon 326 (Ala-GCC/A; standard ambiguity code M for A/C)

^f^ Nucleotide 1170 displaying a synonymous mutation at codon 343 (Gly = GGG/A; standard ambiguity code R for A/G)

^g^ C*yp51* cDNA levels were quantified by the comparative C_T_ method (∆∆C_T_) in the StepOne Plus instrument (Applied Biosystems). Relative expression (relative quantification, RQ) was calculated as 2^-∆∆CT^. Means with the same letter are not significantly different at ∝ = 0.05

### Single nucleotide polymorphism (SNP) genotyping

The genotyping reaction was done for two biological replicates for each isolate. *EnCyp51*-specific forward and reverse primers (FS*cyp51* and RS*cyp51*, Table 2) and Y136/F136-allele-specific TaqMan MGB probes (Probes 1 and 2, Applied Biosystems; Table 2), each labeled with a different reporter dye at the 5′-end, were designed from the *cyp51* consensus sequences using the Custom Taqman Design Tool (Life Technologies). Probe 1 hybridizes to mutant DNA while Probe 2 hybridizes to wild-type DNA. Each 20-μl reaction consisted of 1X Taqman Universal PCR Master Mix (Applied Biosystems), 1X *Encyp51-*customized Taqman CYP51 Genotyping Assay Mix containing primers and probes (Applied Biosystems) and DNA template (5 to 8 ng/μl). Reactions were carried out in the StepOne Plus instrument (Applied Biosystems). After amplification, an end-point plate read was performed in the instrument. The proportion of the mutant and wild-type alleles in the sample was calculated based on fluorescence measurements for each probe by a software algorithm which assigns the corresponding genotype call. For details on the principles behind the protocol, readers are directed to the manufacturer’s manual. Samples were assigned as pure wild-type, pure mutant, or mixed genotype. No signals were generated for other fungal DNA samples (isolated from grape leaves) subjected to this SNP genotyping method indicating the specificity of the primers to *E*. *necator* DNA.

To further test the ability of the SNP genotyping method to detect the Y136F genotypes (TWT and TTT), eight new isolates from each of three different locations (AF, SC, RO) with on-going fungicide efficacy trials were subjected to the method. AF and RO are located in Virginia, and SC in North Carolina. These locations are at a distance of more than a hundred kilometers from each other. The isolates were collected on dates ranging from June to September 2010 from field plots treated with label rates of tebuconazole or fenarimol or not treated, or from treated or untreated potted grapevines placed near such plots. Powdery mildew growth on field leaves was transferred to leaves treated with tebuconazole in the laboratory. Isolates that grew well (profusely sporulating) on leaves treated with a discriminating dose of 10 mg liter^-1^ tebuconazole on the first culture transfer were considered DMI-resistant. These isolates were purified in the same manner as described earlier, then DNA was extracted and analyzed using the SNP genotyping method.

### *Cyp51* overexpression

Representative isolates from each genotype group, four wild-type (TAT) and eight mutants (TTT/TWT), were each grown again on ten grape leaves. Fungal material was scraped from the surface of infected leaves using a sterile spatula into a Lysing Matrix C tube (MP Biomedicals LLC, Solon, OH, USA). Buffer RLC (450 μl; Qiagen, Valencia, CA, USA) was added to the tube, then fungal tissue was disrupted by two consecutive bead-beatings at 4 m s^-1^ for 50 s in the FastPrep-24 instrument (MP Biomedicals LLC, Solon, OH, USA). RNA extraction was completed using the RNEasy Plant Mini Kit (Qiagen) following the manufacturer’s protocol for filamentous fungi. The resulting RNA sample was diluted to 30 ng ml^-1^ and reverse-transcribed using the High Capacity cDNA Kit (Applied Biosystems). Primer information for *Encyp51* (FG*cyp51* and RG*cyp51*) and *Entub* (FG*tub* and RG*tub*) are provided in Table 2. Probes (P*cyp51* and P*tub*; Table 2) labeled with the fluorescent dye FAM at the 5′-end and a non-fluorescent quencher with a minor groove binder (MGB) at the 3′-end were utilized to enable detection of the specific PCR product during amplification. All primers and probes were designed based on our sequences for *Encyp51* and Genbank Accession no. AY074934 for *Entub* [48] using the Custom TaqMan Assay Design Tool (www.lifetechnologies.com), then customized into CYP51 and β-TUB Gene Expression Assay mixes by Applied Biosystems. Reactions (20 μl) contained 1X Taqman Universal PCR Mix (Applied Biosystems), 1X Taqman Gene Expression Assay mix (Applied Biosystems), and 2 μl of the cDNA template. Reactions were done in triplicate, and subjected to the following thermocycling program: a pre-amplification step at 50°C for 2 min, an initial denaturation at 95°C for 10 min, 40 cycles at 95°C for 15 s and 60°C for 1 min. Reverse transcription and gene expression quantitation were performed on two biological replicates for each isolate. C*yp51* cDNA levels were quantified by the comparative C_T_ method (∆∆C_T_) in the StepOne Plus instrument (Applied Biosystems). *Cyp51* expression was normalized to the expression of the *E*. *necator* β-tubulin gene (*Entub*). Relative expression (relative quantification, RQ) was calculated as 2^-∆∆CT^.

### Statistical analysis

Mean RQ values were compared among isolates by analysis of variance. Correlation analysis was done by Pearson’s pairwise comparison of RQ and RF. Statistical analyses were done in JMP Pro v.11 (SAS Institute Inc., Cary, NC, USA).

## Results

### *EnCyp51* sequencing and *Cyp51* genotypes

**Fig 1:**
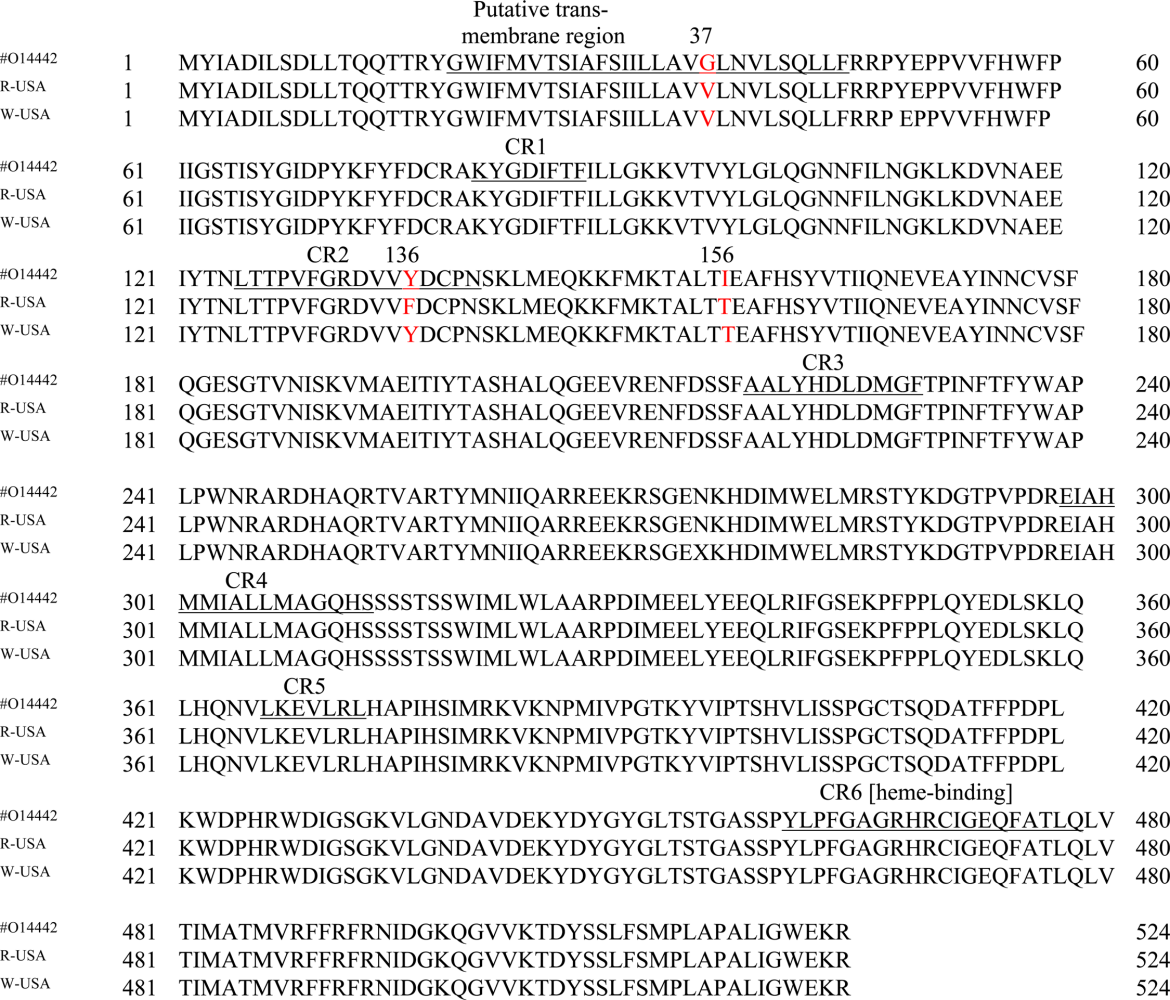
Alignment of the consensus amino acid sequence of CYP51 of *Erysiphe necator* isolates from the United States, with Y136F (R-USA) and without Y136F (W-USA) with the CYP51 (UniProtKB/Swiss-Prot Accession no. O14442) of an *E*. *necator* isolate from Europe, which is based on a Group B isolate [49]. Conserved domains are underlined in the European sequence as proposed by Délye et al. [24]. Amino acids in red indicate a variation from at least one other sequence.

Sequencing of the 1.7 kb-*Encyp51* from 24 isolates revealed the existence of only two alleles (Genbank accession no. KR106192 and KR106193) and two additional silent substitutions (Table 3). BLAST searches showed 99% similarity with *Encyp51* sequences from Europe (GenBank accession no. AF042067, UNU83840, UNU72657) [24, 25, 49] and Australia (GenBank accession no. EF649776, EF649777), indicating not only successful amplification of the *Encyp51* from the US isolates but also its highly conserved nature. The two *Encyp51* allele sequences were translated into 524 amino acids (Fig 1) which were 99.4% identical with the predicted sequence of Délye et al. [24, 49]. Alignment of the predicted amino acid sequence of *Encyp51* of the 24 isolates showed that our isolates had V at position 37 (GV) and T at position 156 (IT), which is characteristic of what in Europe has been variously called Group I or Group A [17, 49].

The allele with the TAT codon corresponding to Y136 was designated wild-type, while that with TTT encoding 136F was designated mutant. The sensitive sub-group consisting of three isolates (BLP1, BLP4, MVP9) possessed the wild-type allele and had EC_50_ values that translated into resistance factors (RF) <3 for tebuconazole, myclobutanil and fenarimol (Table 3). Even though there were only three isolates in the sensitive sub-group, their EC_50_ was in line with sensitive isolates in our region [15]. We therefore used this group as the standard of comparison and their mean EC_50_ as the basis for calculating resistance factors. Two other isolates (SCCP4, PBP1) taken from vineyards had RF similar to the sensitive sub-group and thus were considered DMI-sensitive (Table 3). Another group of isolates (SNP1, SNP3, FH9-1, Table 3) taken from wild grapes were partially sequenced and determined to be wild-type (TAT) also.

In the first sequencing, eleven isolates possessed the TTT genotype (Table 3). Eight of these had RF>3 (corresponding to EC_50_ 0.17–3.5 mg liter^-1^ tebuconazole, 0.73–13.8 mg liter^-1^ myclobutanil, and 0.08–1.3 mg liter^-1^ fenarimol, Table 1) and were weakly resistant or resistant to the three DMIs. The isolate IVP11 had similar resistance profile as the eight isolates except for myclobutanil sensitivity which we were not able to verify because we lost the isolate prior to bioassay. One isolate (GRP15) with TTT genotype was resistant (RF>10) to tebuconazole and fenarimol, but sensitive to myclobutanil (RF<3) (Table 3). The last isolate in the group, IVP3, was classified as sensitive (RF<3) to all three DMIs but still possessed the TTT genotype.

Another isolate, VAHP6, with RF>10 and, therefore, resistant to the three DMIs, possessed the wild-type allele (Table 3). The final set of seven isolates with high resistance factors (highly resistant) revealed a double peak at codon 136, coded as TWT, which indicates the presence of both nucleotides A and T in the same position (Table 3). *Cyp51* sequencing with the first primer set thus revealed three genotypes at the 136^th^ codon for isolates outside the sensitive sub-group (n = 19): (i) TTT genotype (11 of 19), (ii) TWT genotype (4 of 19) and (iii) TAT genotype (4 of 19) (Table 3). Cases such as VAHP6, GRP15 and IVP3 illustrate that the mutant genotypes did not always associate with resistance to the three DMIs.

The new primer set L419/R419 (Table 2) was designed for re-sequencing to confirm the unexpected non-mutated *cyp51* found in four (AMP1, IVP4, JRP3, VAHP6) of the resistant isolates. Three of these (AMP1, IVP4, JRP3) showed a TWT-genotype on the 136^th^ codon using the L/R419 primer set (Table 3). Since the same DNA sample was used, sample contamination could not satisfactorily explain the difference in results between the first and second sequencing for these isolates. We speculated that mutant amplicons were not amplified to the same degree as the wild-type amplicons in the first sequencing. This would result in the TAT genotype obtained with direct sequencing, which gives the consensus or average sequence in a population of amplicons. In fact, upon closer examination of the DNA chromatograms of AMP1, IVP4 and JRP3 from the first sequencing, a second peak for ‘T’ on the 495^th^ nucleotide that was lower than the ‘A’ peak was observed, indicating that mutant amplicons were also present in the PCR product. Why the sequencing with L/R419 did not result in a similar issue may be explained by it being more efficient as a PCR and direct sequencing primer set. VAHP6 was confirmed as wild-type, with the TAT-genotype in the second sequencing. The wild-grape isolates SNP1, SNP3, and FH9-1 were also confirmed as being wild-type on the 495^th^ nucleotide. These three isolates were bio-assayed only for tebuconazole, but the resulting RFs fell within the sensitive range. Overall, among the 24 isolates that were extensively sequenced, those with RF greater than the sensitive range to one or more DMIs, possessed either the TTT or TWT genotype, with the exception of VAHP6. The single change at nucleotide 495, resulting in the Y136F mutation in CYP51, was associated with tebuconazole, myclobutanil and fenarimol resistance. The TWT genotype was associated with mostly high DMI resistance (RF>10) while the TTT genotype was found in isolates with a range of DMI sensitivity, from sensitive (RF<3) to weak resistance (RF = 3–9.9) and resistance (RF>10) (Table 3).

Two other nucleotide polymorphisms resulting in silent mutations in codons 326 and 343 were detected. The change from G to A at nucleotide 1170 in codon 343 was detected in both sensitive and resistant isolates (Table 3). However, the A1119C mutation in codon 326 was detected only in isolates that were highly resistant to DMIs. Interestingly, a double peak (designated ‘R’ for nucleotides G and A) registered for nt 1119 in the same isolates possessing the TWT genotype at codon 136. The DMI-resistant isolate VAHP6 is unique in that it was wild-type for codon 136 but mutant for codons 326 and 343.

### SNP genotypes

All isolates lacking Y136F based on *cyp51* sequencing were assigned a pure wild-type genotype (TAT) with SNP genotyping (Table 3). The wild-grape isolates (SNP1, SNP3, FH9-1) with a wild-type codon based on sequencing with the L419/R419 primer were also confirmed as pure wild-type genotype (TAT) by SNP genotyping. The isolates with a TTT genotype were designated ‘pure’ mutants, whereas the TWT genotype was designated ‘mixed’ (TAT/TTT). VAHP6 was a unique isolate because two independent samples tested by SNP genotyping gave different calls; one was ‘mixed’ and the other ‘pure’. AF, RO and SC isolates subjected to the SNP genotyping method were assigned either a mixed or a pure mutant genotype. Isolates from the AF and RO locations consisted of either only mixed genotype (AF) or pure mutant genotype (RO). The eight isolates from SC consisted of five mixed and three pure mutant genotypes.

Altogether, SNP genotyping results supported the existence of the TTT and TWT genotypes in field isolates of *E*. *necator* and validated the method’s ability in detecting these genotypes. The method was also tested with DNA from grape leaves and from fungal contaminants isolated from grape leaves. No signals were generated from these samples, indicating the specificity of the method for *E*. *necator*.

### Over-expression of *cyp51*

**Fig 2:**
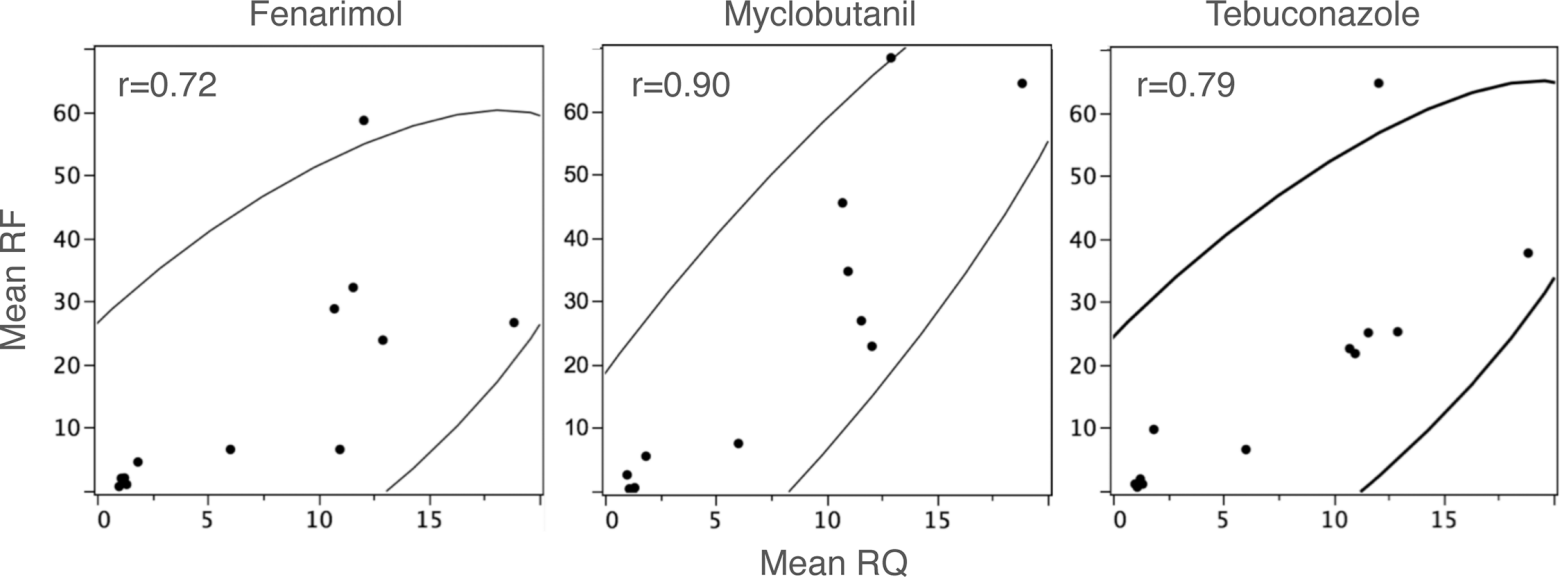
Correlation of the relative quantity (RQ) of *cyp51* (compared to an internal calibrator, a sensitive isolate lacking Y136F, and the *En*β-*tubulin* gene as the normalizer) in 12 *Erysiphe necator* isolates and their sensitivity (RF = resistance factor) to tebuconazole (*P =* 0.0021), myclobutanil (*P*<0.0001) and fenarimol (*P =* 0.0078). Mean resistance factor calculated from at least three independent assays and mean RQ calculated from two independent reverse transcription and gene expression reactions from the same RNA source for each isolate.

A standard curve analysis with a 10-fold dilution series of cDNA was performed on the calibrator isolate (BLP4). PCR efficiency and slopes for *Entub* and *Encyp51* were nearly equal with values of 105% and -3.2 (R^2^ = 0.99), and 106% and -3.18 (R^2^ = 0.99), respectively. Expression values (RQ) were higher in all isolates possessing Y136F (TTT and TWT) than in isolates lacking the mutation. The difference was statistically significant in seven out of nine isolates possessing Y136F (P<0.0001) (Table 3). The highest RQ was generated by JRP4, a TWT-genotype, with a 19-fold increased mRNA level compared to BLP4, our reference DMI-sensitive isolate. The lowest expression level belonged to IVP3 (TTT genotype), which did not significantly differ from the three isolates with TAT-genotype. As a group, the mean expression level of the TAT-genotype (RQ*±*SE = 1.1*±*1.7) was significantly lower than the mutants, with mean RQ of 11.6*±*1.4 and 7.2±1.4 for TWT and TTT, respectively (P<0.002). A significant correlation was also found between cyp51 expression and resistance to the three fungicides (Fig 2): myclobutanil, r = 0.90 (*P*<0.0001); tebuconazole, r = 0.79 (*P* = 0.0021), and fenarimol, r = 0.72 (*P* = 0.0078). All isolates in our gene expression panel that possessed the A1119C mutation exhibited a significantly increased RQ; all of these also had mixed genotype at the 1170th nucleotide (registered as ′R′, Table 3). Three isolates, BXP1A, MdMRP5 and MdMRP7, with the G1170A mutation but not A1119C, exhibited high RQ values as well. The DMI-sensitive isolate MVP9 with the G1170A mutation but not the A1119C had low RQ. This may indicate an association of A1119C but not G1170A with CYP51 over-expression and DMI resistance.

## Discussion and Conclusions

### *En*CYP51 mutations

A high degree of similarity was found among *cyp51* nucleotide sequences of *E*. *necator* isolates from Europe, Australia and North America (Fig 1), which reflects the highly conserved nature of *cyp51* in this species. The sequence of our isolates corresponded with those of European Group A, which fits with the conclusion of Brewer and Milgroom [50] that Group A probably descends from a southeastern US population where A-like isolates [51] are common. Our DMI sensitive isolates tend to be more sensitive [15] than sensitive isolates from the Northeastern US [12], California [11] and Europe [25]. This correlates with the observation that European Group A has higher DMI sensitivity than Group B [52], which is more common in Europe, especially during the middle and later part of the growing season [17], and appears to be the majority or only type found in Australia, South America and western North America [26, 50–53]. Our data apply only to the *cyp51* gene; in other respects our isolates do not necessarily reflect European Group A characteristics. Our isolates are commonly collected late in the season, and commonly found on grape berries and not predominantly on leaves. Ascocarps are commonly observed, and much greater diversity has been documented for southeastern US isolates than for European Group A isolates [51].

One exception to the generally high sensitivity of our sensitive isolates to DMIs is the higher EC_50_ values obtained for myclobutanil in this study. They were higher than those obtained by Colcol et al. [15], and both sets of values were from repeated experiments. An explanation is that the myclobutanil stock used might have lost some of its potency, a possibility that we are unable to verify. Since the RF values are based on tests with the same stock material, the overall conclusions would not change.

Differences in the 37^th^ and 156th amino acid residues between European isolates and our isolates may be an indication of genetic drift or an adaptive response of the populations to changing environmental selection pressures. Amino acid 37 is part of the putative trans-membrane domain, while amino acid 156 is located in the second conserved region (CR2) of CYP51 [24]. The trans-membrane portion on the N-terminal side of the protein may serve as the anchor of *En*CYP51 to the endoplasmic reticulum [25]. Glycine residues in CYP51 may be important to the functional flexibility of the enzyme since this amino acid can fit into hydrophobic and hydrophilic environments with its simple side chain [54]. Valine is a non-polar amino acid and its substitution for glycine can increase hydrophobicity. The 156^th^ amino acid change from isoleucine to threonine may be relevant because it can also decrease hydrophobicity; however, this amino acid is several positions away from the conserved section of the putative substrate-recognition site (CR2 domain) [55]. Mutations in the active sites and other sections of CYP51can influence substrate interactions as shown in the yeast *Candida albicans* [56]. In general, amino acid sequences of the putative substrate-binding regions of CYP51 (CR1 to 6 in *E*. *necator*, Fig 1) are known to be highly conserved across biological kingdoms [57].

The CR2 domain contains the amino acid change Y136F (Fig 1) which has been associated with loss of sensitivity to triadimenol in *E*. *necator* [25]. The corresponding mutation Y137F in other fungi [58] has similar effects in *Mycosphaerella graminicola* [59]. Protein structure modeling of CYP51 in *M*. *graminicola* revealed that the 137^th^ residue is part of the access channel end of the binding pocket. The mutation from tyrosine to phenylalanine expands the heme cavity volume substantially, disrupting triadimenol-binding. The model further showed that loss or gain of resistance to azoles may be due to mutations of certain amino acid residues in the binding pocket, leading to conformational changes in the binding site. The mutation at amino acid 136 of *E*. *necator* may have differential impact on various DMI molecules. In general, the occurrence of Y136F in our isolates with high RFs and its absence in isolates with low RFs support its association with DMI resistance [15, 24]. However, Y136F did not always confer resistance to tebuconazole, myclobutanil and fenarimol based on the finding that two isolates possessed the mutation but were as sensitive to one or more DMIs as the wild-type isolates. This indicates that other mechanisms, acting alone or in combination with the target-site mutation, may be responsible for resistance to DMIs.

### Three genotypes of *cyp51* based on the 136^th^ codon

SNP genotyping supported the existence of two *cyp51* alleles relevant to DMI resistance in *E*. *necator*. This led to the detection of three possible genotypes—TAT, TTT and TWT—based on the 136^th^ codon. The TAT-allele was most commonly present in isolates collected from locations that had probably received little or no exposure to DMIs at the time of sampling (wild grapevines or a new vineyard far from commercial vineyards). The TTT and the TWT genotypes were detected in isolates with low to high resistance to tebuconazole, myclobutanil and fenarimol. Although there was one isolate that could not be resolved by SNP genotyping, the method was able to clarify ambiguities in all other isolates that showed double peaks at the 136^th^ codon in the DNA chromatogram. SNP genotyping produced a ‘mixed’ genotype for these isolates, which were confirmed to be of the TWT genotype after repeat sequencing (e.g. AMP1, IVP4, etc., Table 3). Additional fresh field isolates tested with the SNP genotyping protocol corroborated the existence of both ‘pure’/TTT and ‘mixed’/TWT genotypes. The co-existence of the wild-type and mutant alleles has not been reported elsewhere. Jones et al. [45] did find duplication of *EnCYP51* but all copies within an isolate were of the same allele. The possibility of contamination was considered upon detection of the TWT genotype. To verify the results, two independent DNA samples per isolate were used. Results for the two samples were consistent.

SNP genotyping may be a quicker alternative to sequencing in detecting Y136F and *cyp51* genotypes. Because it is a Taqman-based chemistry and it measures fluorescence signals relative to the amount of the target fragment, it can be optimized to quantify the proportion of the mutation in the population. Such quantitative methods can be applied in field studies such as the quantification done in France, which revealed the Y136F proportion to be low at the national level but increasing in two succeeding years [17]. With large variability in the Y136F percentages among locations in that study, it was suggested that the differences were probably due to local spray programs. We have also tested the SNP genotyping method in another study, revealing co-occurrence of DMI- and QoI-resistance-associated mutations [60]. Our SNP genotyping method has a high degree of specificity because it utilizes *En-*specific probes. We have seen the possibility of a certain genotype dominating the population at a local scale—the mixed genotype in the AF vineyard and the pure mutant genotype in the RO vineyard. Finding out the impact of different DMI fungicides on the evolution of genotypes in field populations may be useful in resistance management.

### *Cyp51* is over-expressed in mutant *E*. *necator*

Since *E*. *necator* is a haploid fungus, our detection of the TWT genotype indicates the presence of at least two copies of the gene. The hypothesis of multiple *cyp51* copies led us to test correlation of resistance level with over-expression. Isolates carrying the mutant genotype, either TTT or TWT, were thus expected to have elevated gene expression. Our results indicated the necessity for the mutated allele for over-expression of CYP51. We demonstrated a 6- to 19-fold increased expression of *cyp51* in seven of nine mutant *E*. *necator* isolates. The remaining two isolates with TTT genotype had low expression, and also low or only moderate resistance to all three DMIs. Since the measurements were made on isolates growing on untreated leaves, the increased expression is considered constitutive.

Correlation analysis showed that our isolates with increased resistance to DMIs tended to have increased *cyp51* constitutive expression, which may explain the quantitative nature of DMI resistance in *E*. *necator*. This was also observed in *Cercospora beticola*, where the highest expression levels of *cyp51* were obtained for highly resistant isolates, while lower expression levels were exhibited by moderately resistant isolates [31]. The expression levels obtained in our study were similar to those observed for epoxiconazole-resistant *Puccinia triticina* [61], fluconazole-resistant *Candida glabrata* [36], DMI-resistant *M*. *graminicola*, myclobutanil-resistant *Venturia inaequalis* [39], propiconazole-resistant *Monilinia fructicola* [40] and fenbuconazole-resistant *Blumeria jaapii* [38]. Expression levels of *cyp51* can be increased more than a hundred fold in triflumizole-resistant *Penicillium digitatum* [37] and DMI-resistant *C*. *beticola* [31, 62]. In most cases, increased expression was due to an insertion in the promoter region of *cyp51*. We were unable to verify this for our isolates because we did not obtain a complete sequence for the promoter region. We conducted PCR reactions using C14/C14R primers of Délye et al. [24] which covered almost the entire 5’ UTR to 3’UTR of *cyp51*. The size of amplicons from mutant and wild-type isolates was indistinguishable. Thus, it is unlikely that insertions or repeats are responsible for increased expression in our *E*. *necator* isolates unless they increase the amplicon size by very little. We found an association of a second mutation A1119C with CYP51 over-expression as reported also by Frenkel et al. [26]. How this mutation would contribute to resistance is unclear based on the knowledge that (i) it does not reside in the promoter region, and (ii) it results in a synonymous mutation. Frenkel’s group speculated that the association could be a coincidence or that the mutation might be impacting mRNA stability.

Many genes involved in the ergosterol pathway, including *cyp51/erg11*, exist in multiple copies in fungi [58, 63, 64]. Duplication of CYP51A in *Aspergillus flavus* and *A*. *oryzae*, and of CYP51B in *A*. *terreus* [63], for example, has been reported. In the human pathogen *C*. *glabrata*, evidence was provided for the association of azole resistance with chromosomal duplication of the *cyp51* gene [36]. Similarly, increased itraconazole resistance was conferred by extra copies of the P-450 14∝-demethylase gene, *pdmA* in *A*. *fumigatus* and *A*. *nidulans* [65]. The resistance level was increased up to 36 times that of wild-type controls. A correlation of copy number of the target gene *ESPS* was also found with resistance to the herbicide glyphosate in populations of several weeds [66].

In *E*. *necator*, increased copy number of *cyp51* was shown to be correlated with gene expression, and may constitute an adaptive mechanism against continuous exposure to DMIs in the field [45]. Duplicate copies can serve to compensate for each other functionally as demonstrated in knock-out experiments of *cyp51* genes in the opportunistic human pathogen *A*. *fumigatus* [67]. Duplicated genes in fungi usually have the same biochemical function but may be differentially regulated at the gene level to bring about ‘partial division of labor’ or sub-functionalization [68]. The finding of three paralogous *CYP51* genes in *Fusarium graminearum* [69] illustrates this and raises a possible explanation for differential sensitivities to DMIs.

The range of *cyp51* expression levels and the occurrence of mixed genotypes among our DMI-resistant *E*. *necator* isolates point to the involvement of copy number variation. Jones et al. [45] provided evidence for CNV of *Encyp51* and its association with Y136F and mRNA transcript levels. Their results indicated that increased copy number of the wild-type allele was rare, suggesting that having multiple copies of the wild-type allele would not confer evolutionary fitness under fungicide selection pressure. We discovered that our isolates highly resistant to the three DMIs not only possessed both wild-type and mutated *cyp51*, but also had high expression levels. The hypothesis that the TWT genotype harbors more copies of the mutant allele than the TTT genotype can be pursued. We are not certain about the importance of maintaining a wild-type copy but this may be beneficial for fitness and survival. The wild-type form may carry a compensatory function, especially when a fitness cost is associated with the mutated copy [45]. Having the wild-type allele may promote optimal sterol biosynthesis, while having the mutant copy or copies serves to ensure sterol biosynthetic capability under selection pressure. In the case of our mutant isolates IVP3 and VAHP1, which were either sensitive or at most moderately resistant to DMIs in leaf bioassays and expressed *cyp51* at wild-type level, sensitivity shifts might have occurred after prolonged culturing on fungicide-free host tissues [46]. Why certain isolates might be more prone to this mechanism than others is unclear. These shifts could be accompanied by loss of some copies of the gene, resulting in low expression of *cyp51*. Evidence of reversion to a sensitive state due to a gradual loss of duplicated chromosomes carrying the *cyp51* gene is found in *C*. *glabrata* [36]. We also found one neutral mutation specific to the TWT genotype. It may have utility as additional molecular marker for monitoring this group in field populations. However, Frenkel et al. [26] proposed that, if the linkage between the mutation and the actual resistance mechanism is only coincidental, the A1119C may become an unreliable marker over time.

In summary, we found two *cyp51* alleles based on the 136^th^ codon corresponding to three genotypes: wild-type (TAT) genotype and two mutant genotypes (TTT and TWT). The mutant genotypes resulted in the Y136F change. However, Y136F only partially explained resistance to tebuconazole, myclobutanil, and fenarimol. *Cyp51* over-expression was associated with the presence of Y136F. Expression levels were higher for isolates with mutant genotypes, and strongly correlated with largely reduced sensitivity to DMIs. Our findings suggest that the mutation may be necessary for resistance, and work in conjunction with copy number variation to confer quantitative resistance in *E*. *necator*. The co-occurrence of cyp51 allelic variants and copy number differences among *E*. *necator* isolates with differential sensitivities and the role that these mechanisms play in DMI resistance are worth further investigation. Cross-resistance was not always observed among the three DMIs tested in this study, which raises another hypothesis, that different forms of CYP51 may also confer a different spectrum of DMI resistance in *E*. *necator*, in a similar manner as the mechanism for paralogous genes discovered in *F*. *graminearum* [69].
